# Patellofemoral arthroplasty-patient demographics and revision causes compared with total and medial unicompartmental knee arthroplasty: long-term follow-up data from the Norwegian Arthroplasty Register

**DOI:** 10.2340/17453674.2025.44593

**Published:** 2025-09-02

**Authors:** Harald Nagelgaard OMENÅS, Einar LINDALEN, Ove Nord FURNES, Anne Marie FENSTAD, Mona BADAWY

**Affiliations:** 1 Coastal Hospital in Hagevik, Orthopaedic Department, Haukeland University Hospital, Hagavik; 2 Department of Orthopaedic Surgery, Lovisenberg Deaconal Hospital, Oslo; 3 The Norwegian Arthroplasty Register, Department of Orthopaedic Surgery, Haukeland University Hospital, Bergen; 4 Department of Clinical Medicine, University of Bergen, Bergen, Norway

## Abstract

**Background and purpose:**

Patellofemoral arthroplasty (PFA) is a rare surgical procedure for isolated patellofemoral osteoarthritis (PFOA). This study compares patient demographics, long-term survival rates, revision risks, and causes of revision in PFA with total knee arthroplasty (TKA) and unicompartmental knee arthroplasty (UKA).

**Methods:**

Data from the Norwegian Arthroplasty Register (NAR) (1994–2022) included 725 PFA, 102,135 TKA, and 14,315 UKA procedures. We used Kaplan–Meier (KM) analysis to calculate implant survival at 2, 5, 10, and 15 years and Cox regression adjusted for confounders to assess revision risks. Revision causes were analyzed for procedures after 2005.

**Results:**

PFA patients were more often female (72%) than TKA (62%) and UKA (51%) patients and had a lower mean age (54.3 for PFA, 69.0 for TKA, and 65.6 for UKA). At 10 years, KM survival was 85% (95% confidence interval [CI] 80.6–88.2) for PFA, 94% (CI 93.8–94.2) for TKA, and 84% (CI 83.6–85.1) for UKA. Among patients < 60 years, KM survival at 10 years was 84% (CI 79.4–88.1) for PFA, 90% (CI 89.3–90.4) for TKA, and 79% (CI 77.1–80.3) for UKA. In patients < 60 years with < 10 years’ follow-up, the adjusted hazard ratios (HR) for revision were 0.9 for TKA and 1.7 for UKA compared with PFA. Adjusted HR for > 10 years’ follow-up showed lower revision risks for TKA with 0.3 and no significant difference for UKA (HR 0.9). Progression of OA was the leading cause of revision in PFA (49%).

**Conclusion:**

PFA was predominantly performed in younger female patients. In patients < 60 years, PFA showed similar 10-year survival to TKA but inferior survival after 15 years. Revision rates for PFA are comparable to UKA but inferior to TKA.

Isolated patellofemoral osteoarthritis (PFOA) is seen in 9% of radiographs of symptomatic knees in individuals over the age of 40 years [1]. PFOA can be primary, or secondary to trochlear dysplasia or posttraumatic [2]. Patellofemoral arthroplasty (PFA) is an alternative to total knee arthroplasty (TKA) for isolated PFOA if nonoperative treatment has failed. Earlier, PFA had inconsistent outcomes but improvements in implants and instrumentation, patient selection, and surgical technique have renewed interest and usage of both unicompartmental knee arthroplasty (UKA) and PFA [3].

High failure rates of PFA have been reported [4], and registry studies show higher revision risks compared with TKA. However, recent studies report promising outcomes, high survival rates, and good patient satisfaction, though with short follow-up. The incidence of patellofemoral dysfunction and failure due to maltracking, instability, and catching has decreased using modern trochlear implant designs [5-7].

Long-term progression of tibiofemoral osteoarthritis remains a primary cause of PFA revision in multiple studies [8-11].

Some studies suggest that UKA is appropriate for about half of knee arthroplasty patients, but its utilization varies from 8% of procedures in the National Joint Registry [12] and 12% in the Norwegian Arthroplasty Register (NAR) [13], to more than 20% in Denmark [14]. Similarly, isolated PFOA affects 9% of patients in a radiological study [1], while PFA represents less than 1% of primary knee replacements across 8 national registries [15].

We aimed to compare demographics, revision risks, and revision causes in PFA, TKA, and UKA over 28 years using Norwegian Arthroplasty Register with 10-year survival as primary endpoint, and 2, 5, and 15 years as secondary endpoints.

## Methods

### Study design and data collection

**Figure 1:**
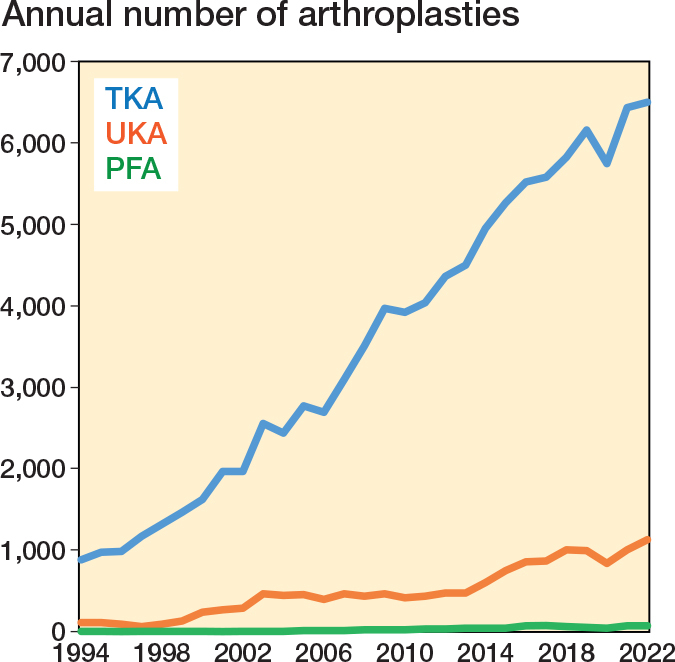
117,175 knee arthroplasties reported to the Norwegian Arthroplasty Register were included in the study from 1994–2022. PFA = patellofemoral arthroplasty, UKA = unicompartmental knee arthroplasty, and TKA = total knee arthroplasty. Hinged and fully constrained TKAs are excluded.

This observational registry study was planned and reported according to the STROBE guidelines [16]. The NAR has collected data on knee arthroplasty since 1994 [17], including procedure type, implant, indication, time to, and reason for revision. We identified 725 PFA, 14,315 UKA, and 102,135 TKA procedures reported between January 1994 and December 2022. Patient and surgical characteristics were analyzed (Figure 1). Accepted indications for PFA are bone-on-bone PFOA on tangential radiographs with intact tibiofemoral joint lines; instability, malalignment, or non-bone-on-bone pain are contraindications [15,18]. Full-thickness patellofemoral cartilage loss is therefore required [19].

In a study from 2024, data from the Norwegian Arthroplasty Register (NAR) and the Norwegian Patient Register (NPR) were used to perform a capture–recapture analysis, which yielded an estimated completeness of 96.8%, consistent with the 97.0% reported by NAR for primary procedures while the completeness for revisions was slightly lower, with a median of 88.9% for knee arthroplasties. The study also showed that NAR has full coverage (all hospitals participate) and that registries with mandatory reporting generally perform best (17).

### Outcomes

Revision reasons were reported by surgeons using predefined checkboxes on the registry form. In 2011, “progression of osteoarthritis” was added as a separate category.

Revision defined as exchange, removal, or addition of components was linked to the index procedure by laterality and unique patient ID; date of deaths came from the National Population Register. Knees were classified as revised, unrevised, or dead. Kaplan–Meier (KM) survival was calculated at 2, 5, 10, and 15 years, using revision for any cause at 10 years as the primary endpoint. Demographic and survival analyses were repeated for patients aged < 60 years.

Hospital volume was based on total PFA procedures from 2005–2022, aligning with consistent ASA and revision data. Hospitals with ≥ 35 PFAs in this period were classified as high-volume centers.

### Statistics

Implant survival was estimated with KM curves with 95% confidence intervals (CI) to 15 years, censoring at death, emigration, or December 31, 2022; median follow-up was calculated using the reverse KM method. Survival differences were tested with the log-rank test. Multivariable Cox models produced hazard ratios (HR) adjusted for sex, age, diagnosis, calendar year, and ASA class, reported relative to PFA for follow up ≤ 10 and > 10 years. Analyses were repeated for all patients and for those aged < 60 years (see Table 3). For Cox regression and revision cause analyses, we restricted data to 2005–2022 based on 2 methodological factors: (i) the number of PFAs performed before 2005 was very small (n = 28), limiting the reliability of early survival estimates; and (ii) ASA classification reporting was available in the registry only from 2005 onward. Proportional hazard assumptions were tested with Schoenfeld residuals. Standardized mean differences (SMDs) assessed covariate balance between implant groups. All tests were 2-sided (α = 0.05) and run in SPSS 29 (IBM Corp, Armonk, NY, USA) and Stata 18 (StataCorp LLC, College Station, TX, USA). A sensitivity analysis was conducted [18]. Missing data was minimal (diagnosis 0.1%, ASA 2.1%). Best- and worst-case imputations for these covariates (see Supplementary Table S2) yielded hazard ratios identical to complete-case models, so further imputation was unnecessary.

### Ethics, funding, and disclosures

The Norwegian Arthroplasty Register has permission from the Norwegian Data Inspectorate to collect patient data based on written consent from the patient (ref 24.1.2021: 16/01622-3/CDG). The authors received no specific funding for this work. No conflicts of interest were declared. Complete disclosure of interest forms according to ICMJE are available on the article page, doi: 10.2340/17453674.2025.44593

## Results

### Patients

**Figure 2:**
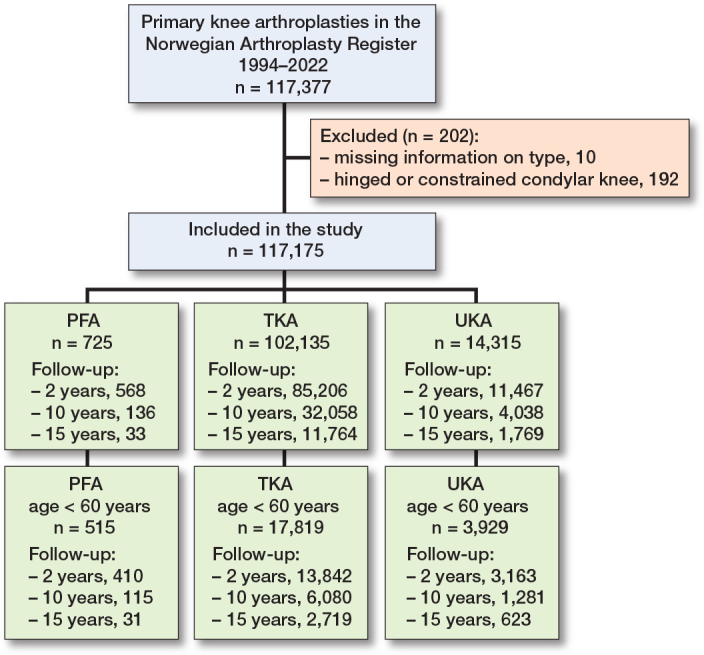
Patient flowchart. For abbreviations, see Figure 1.

Primary knee arthroplasties were included from 1994–2022. 725 PFAs were included, of which 515 were aged < 60 years. 102,134 TKAs were included, of which 17,819 were aged < 60 years and 14,315 UKA were included of which 3,929 were aged < 60 years (Figure 2).

PFA patients were more often female (72%) than TKA (62%) and UKA (51%) patients and had a lower mean age (54.3 for PFA, 69.0 for TKA, and 65.6 for UKA). 71% of PFA patients were under 60 years, compared with 17% of TKA and 28% of UKA patients. Among patients < 60, mean age was 47.9 for PFA, 53.8 for TKA, and 54.1 for UKA. Non-primary OA was more frequent in PFA (29%) than in TKA (12%) and UKA (6.1%); for patients < 60 years, the corresponding values were 36%, 24%, and 11%. Median time to revision and crude revision rates are given in Table 1.

**Table 1:** Demographic data by implant type from 1994–2022. Values are count (%) unless otherwise specified

Item	PFA	TKA	SMD ^a^	UKA	SMD ^a^
**All patients**					
Primary procedures	725	102,138	–	14,317	–
Revisions	80 (11)	5,240 (5.1)	0.22	1,813 (13)	0.05
Median follow-up (IQR)	5.8 (2.9–9.6)	7.2 (3.6–12)	–0.34	6.8 (3.3–12)	–0.22
Median time to revision (IQR)	4.1 (2.3–7.5)	2.1 (0.8–4.9)	0.44	3.4 (1.3–7.9)	0.09
Female sex	521 (72)	63,734 (62)	0.20	7,345 (51)	0.43
Mean age (SD)	54.3 (13)	69.0 (9.6)	–1.32	65.6 (9.3)	–1.02
Under 60 years	515 (71)	17,819 (17)	–1.28	3,929 (27)	–0.97
Diagnosis OA **^b^**	512 (71)	90,086 (88)	0.45	13,444 (94)	0.64
ASA class **^c^**			–0.69		–0.46
1	263 (36)	1,226 (12)		2,720 (19)	
2	422 (58)	68,535 (67)		9,478 (66)	
≥ 3	41 (5.6)	21,245 (21)		2,119 (15)	
Year of surgery			0.48		0.38
1994–2004	28 (3.9)	17,331 (17)		2,284 (16)	
2005–2014	230 (32)	37,794 (37)		4,609 (32)	
2015–2022	467 (64)	47,013 (46)		7,424 (52)	
**Patients aged < 60 years**					
Primary procedures	515	17,819	–	3,929	–
Revisions	64 (12)	1,633 (9.2)	0.11	771 (20)	–0.20
Median follow-up (IQR)	6.2 (3.1-10)	7.8 (3.8–13)	–0.35	8.1 (3.8–14)	–0.31
Median time to revision (IQR)	4.6 (2.5-8.2)	2.7 (1.2–6.3)	0.27	4.2 (1.6–8.9)	0.00
Female sex	385 (75)	10,567 (59)	0.33	2,137 (54)	0.43
Mean age (SD)	47.9 (7.6)	53.8 (5.4)	–0.90	54.1 (4.8)	–0.98
Diagnosis OA **^d^**	328 (64)	13,471 (76)	0.26	3,513 (89)	0.64
ASA class **^e^**			–0.53		–0.32
1	227 (44)	4,152 (23)		1,238 (32)	
2	276 (54)	11,654 (65)		2,377 (61)	
≥ 3	13 (2.5)	1,996 (11)		314 (8.0)	
Year of surgery			0.35		0.42
1994–2004	23 (4.5)	2,402 (13)		664 (17)	
2005–2014	174 (34)	6,794 (38)		1,443 (37)	
2015–2022	318 (62)	8,623 (49)		1,882 (46)	

IQR: interquartile range; PFA: patellofemoral arthroplasty; SD: standard deviation; TKA: total knee arthroplasty; UKA: unicompartmental knee arthroplasty.

a Standardized mean difference (SMD), calculated using stddiff in Stata. Covariates with residual imbalance, as indicated by standardized mean differences (SMD), were adjusted for in the Cox regression analyses.

b Diagnosis missing: all patients n = 152.

c ASA reported since 2005: all patients, n = 95,441

d Diagnosis missing: patients aged < 60 years n = 37.

e ASA reported since 2005: patients aged < 60 years, n = 18,777.

Of the 725 PFAs, the most common implants were NexGen PFJ Gender (58%; Zimmer Biomet, Warsaw, IN, USA) and Journey PFJ (32%; Smith & Nephew, London, UK). Both patellar and trochlear components included both inlay and onlay designs. Older designs (e.g., LCS PFJ) were used before 2005, while modern implants dominated after 2015 (Supplementary Table S1).

### Implant survival

The PFA survival for all patients at 10 years was 85% (CI 80.6–88.2) compared with TKA 94% (CI 93.8–94.2) and UKA 84% (CI 83.6–85.1), respectively (Table 2).

**Table 2:** Kaplan-Meier (KM) survival (%) free of all-cause revision with 95% confidence interval (CI) in parenthesis at 2, 5, 10 and 15 years postoperatively

Arthroplasty	Deaths, n (%)	At risk, n	KM 2-year survival (CI)	At risk, n	KM 5-year survival (CI)	At risk, n	KM 10-year survival (CI)	At risk, n	KM 15-year survival (CI)
**All patients**									
PFA	24 (3.7)	568	97.4 (95.8–98.3)	392	92.0 (89.4–94.0)	136	84.8 (80.6–88.2)	33	72.7 (64.1–79.5)
TKA	25,537 (26)	85,206	97.3 (97.2–97.4)	63,180	95.5 (95.4–95.7)	32,058	94.0 (93.8–94.2)	11,764	92.3 (92.1–92.6)
UKA	2,080 (17)	11,467	95.1 (94.7–95.4)	8,118	91.1 (90.6–91.6)	4,038	84.4 (83.6–85.1)	1,769	78.5 (77.4–79.6)
**Patients aged < 60 years**									
PFA	7 (1.6)	410	97.6 (95.8–98.7)	293	91.9 (88.7–94.2)	115	84.2 (79.4–88.1)	31	70.7 (61.4–78.2)
TKA	1,260 (7.8)	14,842	96.1 (95.8–96.4)	10,997	92.6 (92.2–93.0)	6,080	89.9 (89.3–90.4)	2,719	86.5 (85.7–87.2)
UKA	190 (6.0)	3,165	93.2 (92.3–94.0)	2,290	87.3 (86.1–88.4)	1,281	78.8 (77.1–80.3)	623	70.0 (67.8–72.0)

Log-rank P value < 0.001 for both patient groups.

Deaths after revision (n = 1,829) are not included in number of deaths in the Table.

The survival times of unrevised implants were censored at the last date of observation (December 31, 2022) or at the time of death or emigration.

PFA and TKA had similar survival free of all revisions at 2 years. At 10 years the implant survival deteriorates for both the PFA and UKA compared with TKA, and at 15 years’ follow-up PFA implant survival continues to decrease compared with both UKA and TKA (Figure 2).

**Figure 3:**
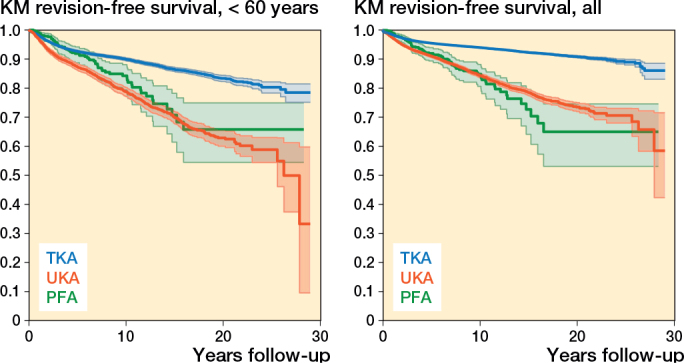
Kaplan–Meier (KM) survival curves for patients < 60 years of age (left panel) and all patients (right panel). For abbreviations, see Figure 1.

Compared with PFA, the adjusted 10-year revision HR was 0.8 (CI 0.6–1.0) for TKA and 1.4 (1.1–1.9) for UKA. Beyond 10 years, PFA showed poorer outcomes: HR 0.3 (CI 0.1–0.6) for TKA and 0.7 (CI 0.4–1.5) for UKA (Table 3). In patients < 60 years, 10-year survival was 84% for PFA, 90% for TKA, and 79% for UKA (Table 3). Adjusted 10-year HRs were 0.9 (CI 0.7–1.2) for TKA and 1.6 (CI 1.1–2.2) for UKA. Beyond 10 years, HRs were 0.3 (CI 0.2–0.6) for TKA and 0.5 (CI 0.3–1.1) for UKA (Figure 3)

**Table 3:** Cox regression analyses showing crude and adjusted hazard ratio (HR) ^a^. HRs are calculated for up to 10 years follow-up (including), and from 10 years and to end of study

	≤10 years of follow up		>10 years of follow up	
	Crude HR	Adjusted HR	Crude HR	Adjusted HR
**All patients**				
PFA	1 (ref)	1 (ref)	1 (ref)	1 (ref)
UKA	1.1 (0.8–1.4)	1.4 (1.1–1.9)	0.3 (0.2–0.6)	0.7 (0.4–1.5)
TKA	0.5 (0.4–0.6)	0.8 (0.6–1.0)	0.1 (0.04–0.1)	0.3 (0.2–0.6)
**Patients aged < 60 years**				
PFA	1 (ref)	1 (ref)	1 (ref)	1 (ref)
UKA	1.4 (1.0–1.9)	1.6 (1.1–2.2)	0.5 (0.3–0.9)	0.5 (0.3–1.2)
TKA	0.7 (0.5–1.0)	0.9 (0.7–1.2)	0.2 (0.1–0.3)	0.3 (0.2–0.6)

a The HRs are adjusted for sex, age, diagnosis, period (analyses > 10 years of follow up do not include period as covariate), and ASA classification. Only procedures from 2005–2022 are included since they constitute 96% (697 of 725 PFA procedures) and registration of ASA class started in 2005.

Procedures from 2005–2022 (697 of 725, 96% of PFAs) showed crude revision rates of 11% for PFA, 5.1% for TKA, and 13% for UKA. Revision for infection was rare in PFA (2.8%) vs TKA (27%) and UKA (8.1%). Hospital volume was limited: 14 centers performed < 5 PFAs, 11 performed 5–30, 3 performed 30–99, and 2 ≥ 100. Centers performing > 35 PFAs had a lower revision risk than lower-volume hospitals.

### Revisions cause

OA progression was the leading cause of PFA revision (49%)—higher than TKA (2.3%) and UKA (23%). Pain alone caused 19% of PFA revisions vs 10% of TKA and 14% of UKA. Loosening and polyethylene wear were less frequent in PFA than in UKA and TKA. No PFA revisions were due to fracture, instability, or dislocation (Table 4). OA progression led to revision at median 5.6 years in PFA and 7.9 years in UKA; pain-only at 4.5 and 3.6 years, respectively.

**Table 4:** Reasons for revision from 2005 ^a^–2022 for PFA, TKA, and UKA. Values are count (%)

Revision causes ^a^	PFA n = 72	TKA n = 3,801	UKA n = 1,224
Infection	2 (2.8)	1,024 (27)	99 (8.1)
Malalignment	5 (6.9)	413 (11)	112 (9.2)
Femoral loosening **^b^**	2 (2.8)	166 (4.4)	111 (9.1)
Tibial loosening **^b^**	0	548 (14)	157 (13)
Patellar loosening	2 (2.8)	7 (0.2)	0
Polyethylene wear	8 (11)	47 (1.2)	44 (3.6)
Bearing dislocation	0	31 (0.8)	56 (4.6)
Patellar dislocation	0	60 (1.6)	1 (0.1)
Instability	0	638 (17)	108 (8.8)
Periprosthetic fracture	0	161 (4.2)	28 (2.3)
Progression of arthrosis	35 (49)	88 (2.3)	281 (23)
Joint stiffness	1 (1.4)	148 (3.9)	7 (0.6)
Pain only	14 (19)	385 (10)	172 (14)
Other	3 (4.2)	176 (4.6)	73 (6.0)
Missing	0	4 (0.1)	3 (0.2)

a Only procedures from 2005–2022 are included as they constitute 96% (697 of 725 PFA procedures).

b A procedure could be registered with both femoral and tibial loosening (95 in the TKA group and 28 in the UKA group).

## Discussion

We aimed to compare PFA with TKA and UKA, focusing on demographics, revision risks and reasons, particularly in younger patients (< 60 years).

Revision rates varied across groups. At 10 years, PFA had a similar revision risk to UKA but higher than TKA in all patients. For patients < 60 years, the PFA revision risk was comparable with TKA but lower than UKA. Beyond 10 years, however, PFA had a threefold higher revision risk than TKA. Progression of OA and pain were the leading causes of PFA revision.

Our study is in accordance with a study by Walker and colleagues, which found that approximately 75% of all patellofemoral arthroplasties were done in women [2], and other studies [6].

In our study, the 10-year survival showed deteriorating results for both PFA and UKA compared with TKA in all patients, probably caused by the increase in revisions due to progression of OA, which is in accordance with earlier studies [19-23]. Only a few randomized controlled trials comparing PFA with TKA for isolated patellofemoral OA have been published, showing that PFA obtained a better overall knee-specific quality of life than patients undergoing TKA throughout the first 2 years after operation for isolated patellofemoral osteoarthritis [6] but after this outcomes for PFA and TKA were similar, and no difference in revision rates was identified [7]. In our study, implant survival declined steadily from 5 to 15 years for both PFA and UKA, most sharply in patients < 60 years. The similar 10-year survival of PFA and TKA, followed by a marked PFA decline from 15 years, probably reflects evolving implant designs, surgical technique, and learning curves; TKA durability has been more consistent. Fewer observations beyond 10 years and rising tibiofemoral OA also widen the confidence intervals.

Surgeons often debate the merits between performing PFA, UKA, or TKA for unicompartmental OA. While TKA eliminates concerns about future OA progression in other compartments, progression remains a common cause of UKA revision. In our UKA group, 23% of revisions were due to OA progression.

Implant design may influence long-term outcomes. In our cohort, the majority of trochlear components were onlay designs, which are associated with improved patellar tracking and fewer complications compared with older inlay designs. The predominance of modern implants like NexGen PFJ and Journey PFJ after 2015 may partly explain the lower rates of instability and dislocation revisions observed in our study. However, due to limited revision events for older designs, statistical comparison of outcomes between implant models was not feasible.

We observed no PFA revisions due to patellar dislocation or instability, which contrasts with earlier literature regarding these complications [24]. This may reflect the impact of improved implant designs and refined surgical technique, including better patellar tracking and component alignment. It is also possible that patients with patellar maltracking or instability were excluded from PFA and instead treated with TKA, reducing the risk of such complications in the PFA cohort. While the absence of these revision causes is reassuring, it should be interpreted cautiously due to the limited number of total revisions in the PFA group.

### Limitations

An important limitation is the inherent difference in patient populations receiving PFA, TKA, and UKA. PFA patients were younger, more often female, and had more secondary OA, reflecting different indications that may confound comparisons. Although we adjusted for age, sex, diagnosis, and ASA in the Cox models, residual confounding is likely. BMI is a known risk factor for OA but was unavailable for this study. Receiving a PFA at a young age with high BMI may increase the risk of future tibiofemoral OA. Additionally, obesity has been linked to poorer clinical outcomes, radiographic outliers, and lower satisfaction in short-term follow-ups [13].

Registry data focuses on revisions, potentially underestimating true implant failure rate, as dissatisfied patients may not undergo revision. Revision procedures also have lower completeness (93%) than primaries (97%), increasing the risk of missing data [16]. Misclassification bias may also occur when surgeons register revision reasons. Bendixen et al. found registry studies report pain more frequently as a revision reason compared with clinical studies, which specify surgical errors more often [11]. However, both types of studies consistently reported progression of OA as the most common cause of revision. Notably, “progression of osteoarthritis” was introduced as a formal revision reason in the NAR in 2011; prior to this, it may have been classified under pain or other causes. Additionally, patient-reported outcomes—such as knee function, pain, satisfaction, and radiographic findings—were not available, excluding functional and satisfaction comparisons between PFA and TKA. Unmeasured factors such as body mass index (BMI), radiographic alignment, and socioeconomic variables could influence both procedure selection and outcomes. Additionally, the lack of patient-reported outcome measures (PROMs) limits the ability to assess functional outcomes, satisfaction, and quality of life—critical elements in the evaluation of arthroplasty success.

In line with previous volume-outcome research, centers performing ≤ 35 patellofemoral arthroplasties annually had higher revision rates, although numbers were small [20,21]. Implant survival did not differ between the common Journey (inlay) and NexGen Gender (onlay) designs.

Although revision causes are prospectively recorded in the Norwegian Arthroplasty Register, misclassification is possible due to differences in surgeon interpretation and changes in the form over time. For example, “progression of osteoarthritis” became a distinct option only in 2011; earlier, it may have been reported as “pain” or as free text under “other”. Registry data were not externally validated in this study, which may introduce reporting bias.

### Conclusion

PFA was a rare procedure and was more often used in younger female patients compared with TKA and UKA. Progression of OA was a major revision cause. Revision rates were similar to UKA but inferior to TKA, especially after 10 years of follow up.

### Supplementary data

Supplementaray Tables S1 and S2 are available as supplementary data on the article page, doi: 10.2340/17453674.2025.44593

## Supplementary Material



## Notes

HNO: conceptualization, methodology, writing the original draft. EL: conceptualization, methodology, review and editing. AMF: data collection, statistics, review and editing. ONF: supervision, methodology, review and editing. MB: supervision, methodology, review and editing.

Handling co-editors: Li Felländer-Tsai and Robin Christensen

*Acta* thanks Per Kjærsgaard-Andersen and Annette W-Dahl for help with peer review of this manuscript.
