# Supplementary material for: Patellofemoral arthroplasty-patient demographics and revision causes compared with total and medial unicompartmental knee arthroplasty: long-term follow-up data from the Norwegian Arthroplasty Register
Source: Acta Orthop. 2025 Sep 2;96:671–6. doi: 10.2340/17453674.2025.44593 (PMC12404100; doi:10.2340/17453674.2025.44593)
Supplement: Supplementary file 1 [file ActaO-96-44593-s1.pdf]

Supplementary data

Supplement Table S1. Distribution of patellofemoral arthroplasty (PFA) implant models by brand, design type, and time period

	Frequency n (%)	Onlay patella	Inlay patella	Onlay trochlea	Inlay trochlea	1994–2004	2005–2014	2015–2022
**Patella Mod III and Patella II** Smith & Nephew	33 (4.6)	X			X	21	12	0
**LINK LUBINUS PATELLA GLIDER** LINK	3 (0.4)	X			X	3	0	0
**LCS PFJ** DePuy Synthes	18 (2.5)	X			X	4	14	0
**Journey PFJ** Smith & Nephew	235 (32)	X	X	X		0	134	101
**Avon-Patellofemoral** Stryker	3 (0.4)	X		X		0	3	0
**Vanguard PFR** Zimmer Biomet	2 (0.3)	X		X		0	2	0
**NexGen PFJ Gender** Zimmer Biomet	419 (58)	X		X		0	61	358
**Sigma Patellofemoral** DePuy Synthes	4 (0.6)	X			X	0	4	0
**iBalance PFJ** Arthrex	8 (1.1)	X		X		0	0	8
**Total**	725					28	230	467

Implant types are categorized by trochlear (onlay vs. inlay) and patellar (onlay vs. inlay) components. Distribution is shown by manufacturer and time period.

Table S2A. Sensitivity analysis from 2005, 10 years' survival, adjusted by imputing best- and worst-case scenario for the values of missing data (all patients)

Item	Best case ≤ 10 years	Worst case ≤ 10 years	Best case > 10 years	Worst case > 10 years
PFA	1 (ref)	1 (ref)	1 (ref)	1 (ref)
UKA	1.45 (1.10–1.92)	1.44 (1.09–1.91)	0.59 (0.31–1.13)	0.58 (0.30–1.12)
TKA	0.78 (0.60–1.02)	0.79 (0.61–1.03)	0.22 (0.12–0.42)	0.23 (0.13–0.42)

ASA missing, n (%): PFA 16 (2.3); UKA 215 (1.8); TKA 1,814 (2.1); total n = 2,045 (2.1).
Diagnosis missing, n (%): PFA 1 (0.1); UKA 13 (0.1); TKA 72 (0.1); total 86 (0.1).
No missing data for sex and age.
Best case: missing ASA = 1 & missing diagnosis = OA.
Worst case: missing ASA = 3 & missing diagnosis = not OA.

Table S2B. Sensitivity analysis from 2005, 10 years' survival, adjusted by imputing best- and worst-case scenario for the values of missing data (patients aged < 60 years)

Item	Best case < 10 years	Worst case < 10 years	Best case > 10 years	Worst case > 10 years
PFA	1 (ref)	1 (ref)	1 (ref)	1 (ref)
UKA	1.62 (1.15–2.26)	1.61 (1.15–2.25)	0.49 (0.24–1.00)	0.49 (0.24–1.00)
TKA	0.92 (0.67–1.25)	0.93 (0.68–1.26)	0.25 (0.13–0.48)	0.26 (0.14–0.48)

ASA missing, n (%): PFA 8 (1.6); UKA 60 (1.8); TKA 330 (2.1); total 398 (2.1).
Diagnosis missing, n (%): PFA 1 (0.2); UKA 5 (0.2); TKA 14 (0.01); total 20 (0.1).
No missing data for sex and age.
Best case: missing ASA = 1 & missing diagnosis = OA.
Worst case: missing ASA = 3 & missing diagnosis = not OA.

Supplementary data

A sensitivity analysis was conducted to assess the potential impact of missing data in the 2 covariates with missing values: diagnosis (reason for operation) and ASA class with 0.1% missing and 2.1% missing respectively. We performed a best-case and a worst-case scenario analysis by imputing the best and worst possible values for these 2 variables. The remaining covariates included in the model were complete (100%). The results from both scenarios were consistent with the main Cox regression analysis, in which cases with missing values were excluded. Given the stability of the resulting estimates and the minimal proportion of missing data, we consider this approach sufficient.
